# TNF‐α interference ameliorates brain damage in neonatal hypoxic–ischemic encephalopathy rats by regulating the expression of NT‐3 and TRKC

**DOI:** 10.1002/ibra.12089

**Published:** 2023-02-19

**Authors:** Yong‐Min Niu, Steven Z. Du, Rong He

**Affiliations:** ^1^ Institute of Neuroscience Kunming Medical University Kunming China; ^2^ Department of Integrative Biology University of Wisconsin‐Madison Madison Wisconsin USA; ^3^ Animal Zoology Department Kunming Medical University Kunming China

**Keywords:** hypoxic–ischemic encephalopathy, neurotrofin‐3, tumor necrosis factor‐α, tyrosine kinase receptor‐C

## Abstract

The aim of this study is to explore the effect of tumor necrosis factor‐α (TNF‐α) inhibition in rats with neonatal hypoxic–ischemic encephalopathy (HIE) and ascertain the relevant signaling pathways. The Zea–Longa score was used to evaluate the neurological function of the rats. ImageJ was used for quantification of the brain edema volume. Triphenyl tetrazolium chloride (TTC) staining of brain tissue was performed 24 h after hypoxic–ischemic (HI) to detect right brain infarction. The expression of TNF‐α was detected by real‐time quantitative polymerase chain reaction (RT‐qPCR). Immunofluorescence staining was used to identify the localization of TNF‐α; Then, the effective shRNA fragment of TNF‐α was used to validate the role of TNF‐α in HIE rats, and the change of neurotrofin‐3 (NT‐3) and tyrosine kinase receptor‐C (TRKC) was examined after TNF‐α‐shRNA lentivirus transfection to determine downstream signaling associated with TNF‐α. Protein interaction analysis was carried out to predict the links among TNF‐α, NT‐3, and TRKC. Cerebral edema volume and infarction increased in the right brain after the HI operation. The Zea–Longa score significantly increased within 24 h after the HI operation. The relative expression of TNF‐α was upregulated after the HI operation. TNF‐α was highly expressed in the right hippocampus post HI through immunofluorescence staining. Bioinformatics analysis found a direct or an indirect link among TNF‐α, NT‐3, and TRKC. Moreover, the interference of TNF‐α increased the expression of NT‐3 and TRKC. TNF‐α interference might alleviate brain injury in HIE by upregulating NT‐3 and TRKC.

## Graphical abstract

Tumor necrosis factor‐α (TNF‐α) interference decreased the overexpression of TNF‐α after the hypoxic–ischemic operation and increased the expression of NT‐3 and tyrosine kinase receptor‐C.

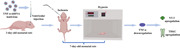

## 1 INTRODUCTION

Hypoxic–ischemic encephalopathy (HIE) is a disease in which damage caused by hypoxic–ischemic (HI) leads to acute brain dysfunction with cell damage and is the leading cause of morbidity and mortality among full‐term born infants throughout the world.^1^, ^2^, ^3^ It increases the rate of death and lifelong disability due to epilepsy, learning disabilities, intellectual deficiency, and brain paralysis.^4^ The morbidity of HIE in developed and developing countries is 1.5 and 10–20 per 1000 live births, respectively.^5^ Worse still, brain damage occurs in five out of every 1000 live births.^6^ Current medical support and hypothermia are standard treatments for moderate to severe HIE, which has been shown to reduce mortality, but 20% of infants with HIE still die.^7^ To date, there are few treatment options for HIE, and the huge financial burden for most families and reduced quality of life caused by HIE are major problems. Therefore, novel and effective treatments must be developed for effective treatment of HIE.

Tumor necrosis factor (TNF), a family of genes involved in inflammation, has strong pro‐inflammatory activity and is one of the key factors in inflammatory bowel disease, spondylitis and brain injury.^8^, ^9^, ^10^, ^11^ TNF‐α, as a cell signaling protein, is one of the cytokines that constitutes the acute phase of the reaction.^12^ Although it can be produced by many other cell types, such as CD4+ lymphocytes, natural killer (NK) cells, neutrophils, mast cells, eosinophilic cells, and neurons, TNF‐α can also be produced primarily by activated macrophages.^13^, ^14^, ^15^ It has been demonstrated that full‐term neonates with HIE have elevated TNF‐α levels and TNF‐α can aggravate the degree of brain injury by reducing neuronal cell viability in the brain.^16^, ^17^ TNF‐α can disrupt the blood–brain barrier and damage cortical cells to aggravate brain injury.^18^, ^19^ Moreover, it has been shown that TNF‐α is a negative factor in HIE and 25 anti‐TNF agents are used to treat inflammatory diseases with TNF dysregulation, but excluding HIE.^20^

In this study, the impact of TNF‐α inhibition and its related molecular mechanism were further investigated, so as to find a new strategy for the treatment of HIE. This is helpful for the application of TNF‐α inhibitors in HIE treatment.

## 2 MATERIALS AND METHODS

### 2.1 Animals

Sprague–Dawley (SD) pregnant rats were obtained from the Animal Center of Kunming Medical University, and 45 7‐day‐old neonatal rats weighing 13–16 g were selected for HI modeling. All animal procedures were approved by the Kunming Medical University Committee on Animal Research (NO. kmmu2019011). Neonatal rats were randomly divided into six groups: sham (*n* = 10), HI‐6 h (*n* = 5), HI‐12 h (*n* = 5), HI‐24 h (*n* = 15), negative control (NC, *n* = 5), and TNF‐α‐shRNA (*n* = 5) groups.

### 2.2 Establishment of neonatal rat HI models

Seven‐day‐old SD rats were weighed and numbered before administration of 3% isoflurane anesthesia. Before the operation, the hypoxia chamber was set up with a temperature of 37°C and humidity of 50%–80%. A 0.5 cm skin incision was made in the midline of the neck with scissors to expose the right common carotid artery (CCA), which was blocked by an electrocautery device (Spring Medical Beauty Equipment Co, Ltd). After recovering for 1 h beside their mothers, the postoperative rats were placed into the hypoxia chamber with 8% O_2_ and 92% N_2_ (flow velocity 3 L/min) for 2 h after waiting with their mothers for 1 h, then taken out and placed together with their mothers. For anesthetized rats in the sham group, only the right CCA at the skin incision was separated without right CCA ligation and oxygen deficiency.

### 2.3 Zea–Longa score

Over six periods, pre‐operation, and 0, 4, 8, 12, and 24 h after the HI operation, the Zea–Longa score was used to assess the severity of nerve injury and to determine the success of model construction, and the scoring criteria were quintile: 0, normal without nerve injury; 1, the forelimb on the contralateral side of the injury could not be extended; 2, tending to rotate on the contralateral side of the injury when crawling; 3, dumping on the contralateral side of the injury when crawling; and 4, could not walk and lost consciousness.

### 2.4 Sample acquisition

Rats were anesthetized and the thoracic cavity was rapidly opened to expose the heart 24 h after surgery. Then, the tip of the 50‐ml syringe was inserted into the ascending aorta from the apex and perfused with 0.9% saline. Afterwards, brain tissues were quickly removed and frozen at −20°C for 10 min for triphenyl tetrazolium chloride (TTC) staining. Fresh samples of the hippocampus, cortex, lung, and heart were directly placed in RNase free EP tubes and frozen at −80°C for real‐time quantitative polymerase chain reaction (RT‐qPCR). For immunofluorescence staining, brain tissues were isolated, fixed with 4% paraformaldehyde (4°C, pH = 7.4) at 4°C for 5 h, and stored in 0.1 Mol (M) phosphate buffer containing 30% sucrose at 4°C for 72 h. Finally, brain tissues were embedded in paraffin and serially cut into 5 μm thick slices.

### 2.5 TTC staining

The cryostat was used to cut the frozen brain tissue into brain sections with a thickness of about 2 mm, which were placed in a tinfoil paper‐coated culture dish for cassette operation. Subsequently, an appropriate amount of 2% 2,3,5‐triphenyltetrazolium chlorides (Sigma Co.) was added to just immerse the sections, which was placed in a 37°C incubator for incubation for 30 min after covering the cassette lid. Then, the sections were washed with phosphate buffer saline (PBS) and fixed with 4% paraformaldehyde. The normal brain tissues were stained with bright red; if there is the infarction area, the area will not be stained and is pale due to decreased dehydrogenase activity. The images were imported into ImageJ software (version 1.52a, NIH) to quantify the cerebral infarction ratio and brain swelling.

### 2.6 Immunofluorescence staining

Sections of brain tissue harvested in vivo were deparaffinized and tissue was encircled by a circle drawn with an immunohistochemical pen. Slices were rinsed three times with 0.01 M PBS (pH 7.4) for 5 min each at room temperature and subsequently preincubated with 0.3% Triton X‐100 and 0.1% bovine serum albumin (BSA) for 1 h. After that, the prepared mixture of the TNF‐α primary antibody (1:500, rabbit, Santacruz) was added to the sections and incubated overnight at 4°C. Tissue sections were rinsed three times for 5 min with 0.01 M PBS containing 0.1% Tween 20. Afterwards, sections were incubated with DayLight 594 antibody (1:200, anti‐rabbit, Jackson) and counterstained for nuclei with DAPI. Finally, fluorescence microscope (DM4000B, Leica) at ×200 was used to acquire image of the hippocampus to observe the range of DAPI and TNF‐α.

### 2.7 Bioinformatics prediction

At the String website (https://cn.string-db.org/), TNF‐α, NT‐3, and TRKC were input into the multiple proteins mode, *Rattus norvegicus* was selected in organisms, and search was clicked to get the protein interaction network image which was exported to PNG format.

### 2.8 Ventricular injection of effective TNF‐α‐shRNA lentivirus

To determine the effect of TNF‐α on HIE rats, 3‐day‐old neonatal rats were anesthetized with 1.5%–2% isoflurane in oxygen‐enriched air. Five microliters (2 × 10^8^/ml) of TNF‐α‐shRNA (GCCCGTAGCCCACGTCGTCGTA) lentivirus (provided by RiboBio) was slowly injected into the right lateral ventricles at 1 μl/min under stereotactic guidance in the TNF‐α‐shRNA group (Digital stereotaxis Instrument with Fine Drive, My Neuro lab; coordinates: *x* = ± 0.5, *y* = +1.0, *z* = + 2.5 mm relative to bregma) and saline in the NC group.^21^ After 3 min, the tip was slowly pulled out and the skin was closed. To detect the inhibitory effect of the TNF‐α‐shRNA lentivirus injection, the expression of TNF‐α was determined via RT‐qPCR.

### 2.9 RT‐qPCR

The entire RNA of the fresh cortex, hippocampus, lung, and heart at 6, 12, and 24 h after HI modeling were isolated by RNAisoplus. Then, using the Revert Aid First Strand cDNA Synthesis Kit (Thermo Fisher Scientific) and following the manufacturer's instructions, β‐actin was amplified by reverse transcription of mRNA into complementary DNA (cDNA) using a RT‐qPCR apparatus (CFX‐96, Bio‐rad). The primer sequences synthesized by Shuoqing Biotechnology Company were as follows: TNF‐α: forward, GTTGGACCAATCATAGGCGC; reverse, CAATGTCG ATCACATGCACCA; NT‐3: forward, ACCGAACTCGAGTCCACCT; reverse, TGGAATTCTGACCTGGTGGC; TRKC: forward, GTCTGCAGCAAGACTGAGAT, reverse, CCAGTTCTCTATGTGTCTGG; β‐actin: forward, GAAGATCAAGATCATTGCTCCT; and reverse, TACTCCTGCTTGCTGATCCA. The protocol of the variable‐temperature heater was followed to carry out PCR: first, 95°C for 5 min, followed by 40 cycles of 95°C for 10 s, 51°C for 10 s, and 60°C for 20 s. The data were analyzed using the comparative critical threshold method with normalization of the β‐actin value to 2^−^^ΔΔCt^, which was used to determine the expression levels of TNF‐α, NT‐3, and TRKC.

### 2.10 Statistical analysis

Mean ± standard deviation of the mean (SEM) and SPSS 25 (IBM Corp) were adopted to describe and analyze the experimental data, respectively. The normality test of numerical variables showed that they obeyed a normal distribution, so we used an independent‐samples *t*‐test for comparison between two groups and one‐way analysis of variance (ANOVA) for comparison between multiple groups. The cerebral infarction ratio and brain swelling were analyzed using an independent‐samples *t*‐test and other data using one‐way ANOVA methods according to variables. *p* < 0.05 was considered to indicate statistical significance.

## 3 RESULTS

### 3.1 Successful establishment of an animal model of HIE

**Figure 1:**
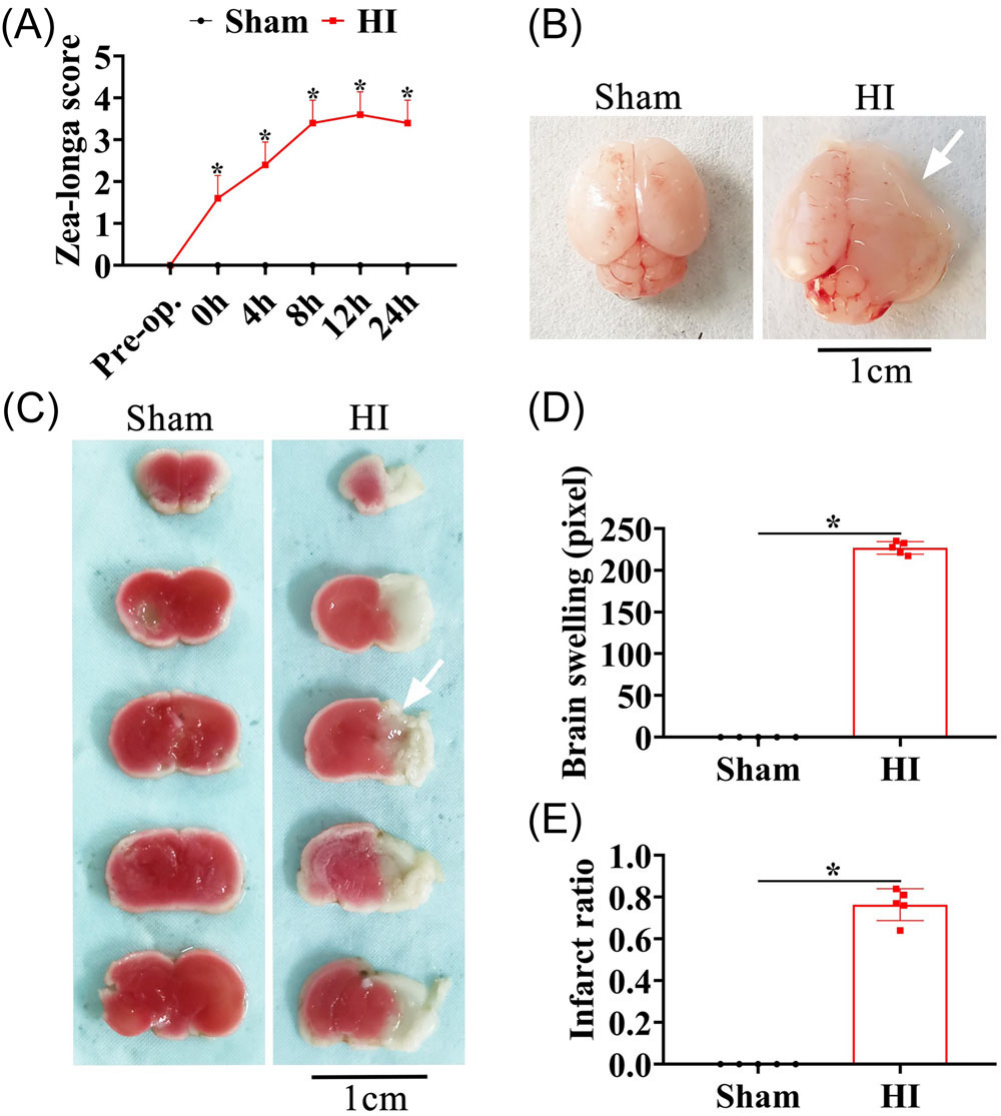
Changes in the Zea–Longa score, edema, and infarction in the right brain after the HI operation. (A) Changes in the Zea–Longa score after the HI operation compared to the sham group. (B, D) Changes in right brain edema after the HI operation compared to the sham group. (C, E) Changes in right cerebral infarction after the HI operation compared with the sham group. Data were described as mean ± SEM. HI, hypooxic‐ischemic; h, hours. *p < 0.05. [Color figure can be viewed at wileyonlinelibrary.com]

The Zea–Longa score in HIE injured rats increased compared to the sham group at 0, 4, 8, 12, and 24 h after surgery (Figure 1A, *p* < 0.05). The edema volume of the right brain of rats after the HI operation clearly increased compared with that of the sham group (Figure 1B,D, *p* < 0.05). The results of TTC staining of brain sections from SD rats showed that the volume of right cerebral infarction significantly increased after the HI operation (Figure 1C,E, *p* < 0.05). The above results showed that the neonatal rat HI model was successfully constructed.

### 3.2 Expression of TNF‐α upregulated in the lung, hippocampus, and cortex after the HI operation

**Figure 2:**
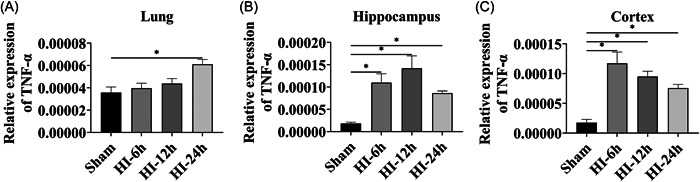
Relative expression of TNF‐α after HI operation at 6 h, 12 h, and 24 h. (A‐C) The relative expression of TNF‐α was determined by RT‐qPCR after the HI operation in the lung (A), hippocampus (B), and cortex (C). Data were described as mean ± SEM. HI, hypoxic‐ischemic; h, hours. *p < 0.05.

We examined the expression of the inflammatory factor TNF‐α following HIE. The results of RT‐qPCR showed that the relative expression level of TNF‐α showed clear upregulation after HI 6, 12, and 24 h in the hippocampus and the cortex, and also 24 h post HI in the lung when compared with the sham group (Figure 2A–C, *p* < 0.05), which indicated a severe inflammatory response after the HI operation.

### 3.3 Localization of TNF‐α in the hippocampus

**Figure 3:**
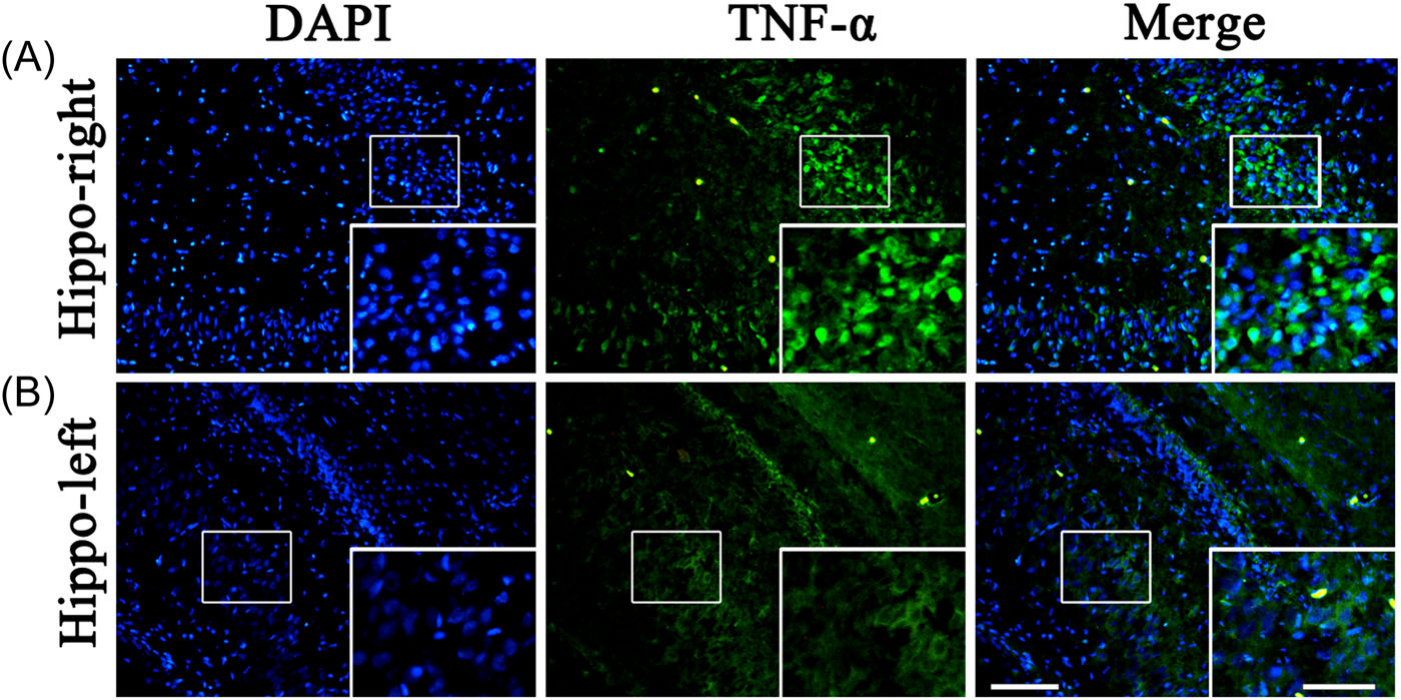
Immunofluorescent of TNF‐α in the hippocampus. (A, B) The localization of TNF‐α was detected by immunofluorescence in the hippocampus. Sections were stained with DAPI (blue, the first panel) to show the nucleus of all cells, TNF‐α (green, the second panel), and the merged images (the last panel). Scale bars, 100 μm, 50 μm for the enlarged area. Hippo‐right, hippocampus right; Hippo‐left, hippocampus left. [Color figure can be viewed at wileyonlinelibrary.com]

We observed changes of TNF‐α in the left and right hippocampus. The results of immunofluorescence staining indicated that TNF‐α was expressed in the hippocampus, and its expression was higher in the right hippocampus (Figure 3).

### 3.4 Correlation analysis between TNF‐α, NT‐3, and TRKC

**Figure 4:**
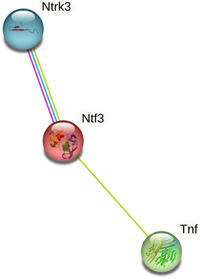
Protein interaction analysis of TNF‐α, NT‐3 and TRKC. The red lines and the blue line represent known interactions between proteins that have been experimentally determined and from curated databases, respectively; the green lines represent text mining. [Color figure can be viewed at wileyonlinelibrary.com]

To further confirm the effect of TNF‐α in brain injury following HIE, we selected NT‐3 (encoded by the neurotrophin 3 [Ntf3]) and TRKC (encoded by the neurotrophin receptor tyrosine kinase [Ntrk3]), which are closely related to brain injury. Protein–protein interaction analysis indicated a direct or an indirect link among TNF‐α, NT‐3, and TRKC (Figure 4). Therefore, it is likely that TNF‐α plays a role in HIE through potential mechanisms that directly affect NT‐3 and indirectly affect TRKC.

### 3.5 Relative expression of NT‐3, and TRKC upregulated after TNF‐α interference

**Figure 5:**
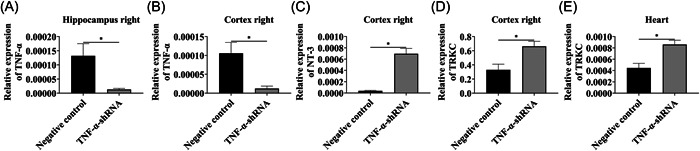
Effect on the expression of TNF‐α, NT‐3 and TRKC after interfering TNF‐α in vivo. (A) Compared with the NC group, the relative expression changes of TNF‐α in the right hippocampus in the TNF‐α‐shRNA group. (B, C, D) Compared with the NC group, the relative expression changes of TNF‐α, NT‐3, and TRKC in the right cortex in the TNF‐α‐shRNA group. (E) Relative expression changes of TRKC in the heart compared with the NC group. Data were described as mean ± SEM. *p < 0.05.

We verified whether TNF‐α‐shRNA lentivirus transfection was successful and detected expression of TNF‐α, NT‐3, and TRKC. TNF‐α expression in the cortex and hippocampus of the TNF‐α‐shRNA group was significantly lower than that of the NC group after TNF‐α interference, indicating successful ventricular injection of effective TNF‐α‐shRNA lentivirus (Figure 5A,B, *p* < 0.05). The expression levels of NT‐3 in cortex and TRKC in cortex and heart were upregulated after TNF‐α interference compared with the NC group in cortex and heart (Figure 5C,D,E, *p* < 0.05).

## 4 DISCUSSION

In this study, we investigated the relationship between TNF‐α and NT‐3, TRKC in neonatal HI rats, and the results showed that edema and increased infarct volume in the right brain occurred after HI modeling. Moreover, the relative expression level of TNF‐α was increased in the neonatal rats after HI modeling. Furthermore, the expression of NT‐3 and TRKC was found to be increased after TNF‐α interference, which indicated that TNF‐α interference ameliorates brain damage in neonatal hypoxic–ischemic encephalopathy rats via overexpression of NT‐3 and TRKC.

### 4.1 Increased cerebral infarction and edema after the HI operation

We found that the infarct volume and edema in the right brain after the HI operation were markedly increased compared to that in the sham group, which has been confirmed by previous studies.^22^, ^23^ Breakdown of the blood–brain barrier leads to systemic leukocyte infiltration into the brain parenchyma and inflammation, further enhancing the formation of cerebral edema, which peaks at 24–48 h after the HI operation.^24^ Dysregulation of the sodium and potassium pump causes additional calcium influx into cells, exacerbating negative effects such as ischemia and microvascular injury, leading to insufficient blood supply and an increase in cerebral infarction areas.^25^ Most brain tissue structures are paralyzed in functional areas due to brain edema and infarction injury caused by HI, which seriously endangers the life and health of newborns.

### 4.2 Upregulation of NT‐3 and TRKC after silencing TNF‐α

In our study, it was discovered that the expression level of TNF‐α was increased in the neonatal HI rats, which was consistent with previous studies.^26^, ^27^ TNF‐α is one of the key factors causing inflammation in a variety of diseases and can promote oxidative stress, necrosis, and apoptosis at the site of injury. Cerebral ischemia can induce an increase in both mRNA and protein expression levels of TNF‐α in the lung tissues of rats with lung injury.^28^ It has also been reported that the expression of TNF‐α in the hippocampus, cortex, and lung tissues gradually increased after brain damage.^29^, ^30^ This indicates that TNF‐α is one of the key factors in the pathomechanism of HIE. Some studies have found that the expression of proinflammatory factor TNF‐α is upregulated in the hippocampus after nerve injury, while the expression of the brain‐derived neurotrophic factor is downregulated, which shows a negative relationship.^31^ Moreover, TNF‐α was significantly increased in ischemic retinal injury, while NT‐3 expression was continuously and significantly downregulated, and there was a negative correlation between the two that is not reported in HIE.^32^ Then, after silencing TNF‐α, the results showed that NT‐3 and TRKC were upregulated in the HI condition. NT‐3 is a protein encoded by the NTF3 gene in humans and synthesized in various tissues, and the protein encoded by the NT‐3 gene is a neurotrophic factor in the family of neurotrophic protein NGF.^33^, ^34^ Downregulation of cortical NT‐3 expression has been reported in brain‐related diseases.^35^ NT‐3 could inhibit glial cell differentiation, improve the microenvironment of the lesion area, differentiate and survive existing neurons, promote endogenous nerve and brain tissue regeneration in previous study.^36^ TRKC, a membrane‐bound receptor for NT‐3, is encoded by the Ntrk3 gene and mainly found in neurons, microglia, astrocytes, and endothelial cells in the central nervous system. Also, it can mediate the neuroprotective function of NT‐3 and regulate neuronal survival, morphology, excitability, and neurite outgrowth by activating TRKC.^37^, ^38^ Downregulation of cortical TRKC expression has been reported in brain‐related diseases.^39^ Some studies have found that the inflammatory response produced by brain injury can affect cardiac function or even cause death, TRKC mRNA expression is significant in the heart, and NT‐3 can reduce cardiomyocyte apoptosis to ameliorate the damage caused by myocardial ischemia/reperfusion.^40^, ^41^ It has been shown that interaction of NT‐3 with TRKC can promote synaptogenesis of neural stem cell (NSC)‐derived neurons in spinal cord injury.^42^ Similarly, it has been shown that the NT‐3/TRKC signaling pathway can ameliorate manganese‐induced neurotoxic effects and increase the survival of neurons.^43^ HIE is associated with heart rate disorders, and studies have found that the severity of neonatal hypoxic‐ischemic encephalopathy can be reflected by heart rate variability (HRV).^44^ NT‐3 and TRKC is associated with cardiac development and can maintain cardiovascular balance, which maybe improve the condition of HRV reduction caused by HIE.^45^, ^46^ Therefore, by silencing TNF‐α, NT‐3 and TRKC can be upregulated to promote the recovery of neurological function and other pathological lesions after HI injury, which maybe improve the mental retardation and movement disorders caused by brain injury.

## 5 CONCLUSION

In summary, TNF‐α interference may ameliorate brain dysfunction due to HIE by upregulating NT‐3 and TRKC. At present, there are very few reports on the application of TRKC in HIE. Therefore, this provided further evidence to reveal the molecular mechanism of the action of TNF‐α in neuronal injury after an HI operation. These findings maybe make TNF‐α inhibitor a new way to ameliorate nerve damage caused by HIE.

## AUTHOR CONTRIBUTIONS

Rong He contributed the central idea and conceived the entire experiment. Yong‐Min Niu analyzed the data and wrote the first draft of the manuscript. Steven Z. Du helped to sort out ideas and provided guidance on writing the article.

## CONFLICT OF INTEREST STATEMENT

There authors declare no conflict of interest.

## ETHICS STATEMENT

The animal study protocol was legally approved by the Animal Care & Welfare Committee of Kunming Medical University (NO. kmmu2019011). All experiments conformed to the Guide for the Care and Use of Laboratory Animals published by the US National Institutes of Health.

## Data Availability

The data that support the findings of this study are available on request from the corresponding author. The data are not publicly available due to privacy or ethical restrictions.

## References

[1] Yıldız EP , Ekici B , Tatlı B . Neonatal hypoxic ischemic encephalopathy: an update on disease pathogenesis and treatment. Expert Rev Neurother. 2017;17(5):449‐459. 10.1080/14737175.2017.1259567 27830959

[2] Rodríguez M , Valez V , Cimarra C , Blasina F , Radi R . Hypoxic‐ischemic encephalopathy and mitochondrial dysfunction: facts, unknowns, and challenges. Antioxid Redox Signal. 2020;33(4):247‐262. 10.1089/ars.2020.8093 32295425

[3] Douglas‐Escobar M , Weiss MD . Hypoxic‐ischemic encephalopathy: a review for the clinician. JAMA Pediatr. 2015;169(4):397‐403. 10.1001/jamapediatrics.2014.3269 25685948

[4] Zhao M , Zhu P , Fujino M , et al. Oxidative stress in hypoxic‐ischemic encephalopathy: molecular mechanisms and therapeutic strategies. Int J Mol Sci. 2016;17(12):2078. 10.3390/ijms17122078 27973415 PMC5187878

[5] Greco P , Nencini G , Piva I , et al. Pathophysiology of hypoxic‐ischemic encephalopathy: a review of the past and a view on the future. Acta Neurol Belg. 2020;120(2):277‐288. 10.1007/s13760-020-01308-3 32112349

[6] Hamdy N , Eide S , Sun HS , Feng ZP . Animal models for neonatal brain injury induced by hypoxic ischemic conditions in rodents. Exp Neurol. 2020;334:113457. 10.1016/j.expneurol.2020.113457 32889009

[7] Lyu H , Sun DM , Ng CP , et al. A new hypoxic ischemic encephalopathy model in neonatal rats. Heliyon. 2021;7(12):e08646. 10.1016/j.heliyon.2021.e08646 35024484 PMC8723992

[8] van Loo G , Bertrand MJM . Death by TNF: a road to inflammation. Nat Rev Immunol. 2022:1‐15. 10.1038/s41577-022-00792-3 36380021 PMC9665039

[9] Brüner M , Dige A , Loft AG , et al. Spondylitis‐psoriasis‐enthesitis‐enterocolitis‐dactylitis‐uveitis‐peripheral synovitis (SPEED‐UP) treatment. Autoimmun Rev. 2021;20(2):102731. 10.1016/j.autrev.2020.102731 33326852

[10] Garg C , Seo JH , Ramachandran J , Loh JM , Calderon F , Contreras JE . Trovafloxacin attenuates neuroinflammation and improves outcome after traumatic brain injury in mice. J Neuroinflammation. 2018;15(1):42. 10.1186/s12974-018-1069-9 29439712 PMC5812039

[11] Zelová H , Hošek J . TNF‐α signalling and inflammation: interactions between old acquaintances. Inflamm Res. 2013;62(7):641‐651. 10.1007/s00011-013-0633-0 23685857

[12] Ko KI , Coimbra LS , Tian C , et al. Diabetes reduces mesenchymal stem cells in fracture healing through a TNFα‐mediated mechanism. Diabetologia. 2015;58(3):633‐642. 10.1007/s00125-014-3470-y 25563724 PMC4346353

[13] Tay C , Liu YH , Hosseini H , et al. B‐cell‐specific depletion of tumour necrosis factor alpha inhibits atherosclerosis development and plaque vulnerability to rupture by reducing cell death and inflammation. Cardiovasc Res. 2016;111(4):385‐397. 10.1093/cvr/cvw186 27492217

[14] Junaid A , Schoeman J , Yang W , et al. Metabolic response of blood vessels to TNFα. eLife. 2020;9:9. 10.7554/eLife.54754 PMC7476757 32749215

[15] Meyer SU , Thirion C , Polesskaya A , et al. TNF‐α and IGF1 modify the microRNA signature in skeletal muscle cell differentiation. Cell Commun Signaling. 2015;13:4. 10.1186/s12964-015-0083-0 PMC4325962 25630602

[16] Massaro AN , Wu YW , Bammler TK , et al. Plasma biomarkers of brain injury in neonatal hypoxic‐ischemic encephalopathy. J Pediatr. 2018;194:67‐75. 10.1016/j.jpeds.2017.10.060 29478510

[17] Shao X , Yang X , Shen J , et al. TNF‐α‐induced p53 activation induces apoptosis in neurological injury. J Cell Mol Med. 2020;24(12):6796‐6803. 10.1111/jcmm.15333 32344470 PMC7299703

[18] Wang K , Wang H , Lou W , et al. IP‐10 promotes blood‐brain barrier damage by inducing tumor necrosis factor alpha production in Japanese encephalitis. Front Immunol. 2018;9:1148. 10.3389/fimmu.2018.01148 29910805 PMC5992377

[19] Wang JY , Huang YN , Chiu CC , et al. Pomalidomide mitigates neuronal loss, neuroinflammation, and behavioral impairments induced by traumatic brain injury in rat. J Neuroinflammation. 2016;13(1):168. 10.1186/s12974-016-0631-6 27353053 PMC4924242

[20] Jarosz‐Griffiths HH , Holbrook J , Lara‐Reyna S , McDermott MF . TNF receptor signalling in autoinflammatory diseases. Int Immunol. 2019;31(10):639‐648. 10.1093/intimm/dxz024 30838383

[21] Xiong LL , Xue LL , Du RL , et al. Vi4‐miR‐185‐5p‐Igfbp3 network protects the brain from neonatal hypoxic ischemic injury via promoting neuron survival and suppressing the cell apoptosis. Front Cell Dev Biol. 2020;8:529544. 10.3389/fcell.2020.529544 33262982 PMC7688014

[22] Xu LX , Lv Y , Li YH , et al. Melatonin alleviates brain and peripheral tissue edema in a neonatal rat model of hypoxic‐ischemic brain damage: the involvement of edema related proteins. BMC Pediatr. 2017;17(1):90. 10.1186/s12887-017-0824-x 28351378 PMC5371222

[23] Huang A , Jia L . Crocin enhances hypothermia therapy in hypoxic ischemia‐induced brain injury in mice. Acta Neurol Belg. 2021;121(2):429‐436. 10.1007/s13760-019-01198-0 31367946

[24] Ma Q , Dasgupta C , Li Y , Huang L , Zhang L . MicroRNA‐210 suppresses junction proteins and disrupts blood‐brain barrier integrity in neonatal rat hypoxic‐ischemic brain injury. Int J Mol Sci. 2017;18(7):1356. 10.3390/ijms18071356 28672801 PMC5535849

[25] Allen KA , Brandon DH . Hypoxic ischemic encephalopathy: pathophysiology and experimental treatments. Newborn Infant Nurs Rev. 2011;11(3):125‐133. 10.1053/j.nainr.2011.07.004 21927583 PMC3171747

[26] Borjini N , Sivilia S , Giuliani A , et al. Potential biomarkers for neuroinflammation and neurodegeneration at short and long term after neonatal hypoxic‐ischemic insult in rat. J Neuroinflammation. 2019;16(1):194. 10.1186/s12974-019-1595-0 31660990 PMC6819609

[27] Li SJ , Liu W , Wang JL , et al. The role of TNF‐α, IL‐6, IL‐10, and GDNF in neuronal apoptosis in neonatal rat with hypoxic‐ischemic encephalopathy. Eur Rev Med Pharmacol Sci. 2014;18(6):905‐909. 24706318

[28] Liao F , Dan QQ , Du RF , Li JT , Zhang YH . Expression of TNF‐alpha in lung tissue of rats with lung injury induced by brain ischemia. Sichuan Da Xue Xue Bao Yi Xue Ban. 2012;43(6):914‐917. 23387227

[29] Yan Z , Sun XL , Hu YL , Liu M . Different expression of TNF‐alpha in brain and peripheral organs after cerebral contusion of rats. Fa Yi Xue Za Zhi. 2012;28(4):261‐264. 23033663

[30] Chen S , Dong Z , Cheng M , et al. Homocysteine exaggerates microglia activation and neuroinflammation through microglia localized STAT3 overactivation following ischemic stroke. J Neuroinflammation. 2017;14(1):187. 10.1186/s12974-017-0963-x 28923114 PMC5604224

[31] Liu Y , Zhou LJ , Wang J , et al. TNF‐α differentially regulates synaptic plasticity in the hippocampus and spinal cord by microglia‐dependent mechanisms after peripheral nerve injury. J Neurosci. 2017;37(4):871‐881. 10.1523/jneurosci.2235-16.2016 28123022 PMC5296781

[32] Guo XJ , Tian XS , Ruan Z , et al. Dysregulation of neurotrophic and inflammatory systems accompanied by decreased CREB signaling in ischemic rat retina. Exp Eye Res. 2014;125:156‐163. 10.1016/j.exer.2014.06.003 24954538

[33] Zhang J , Shi Q , Yang P , et al. Neuroprotection of neurotrophin‐3 against focal cerebral ischemia/reperfusion injury is regulated by hypoxia‐responsive element in rats. Neuroscience. 2012;222:1‐9. 10.1016/j.neuroscience.2012.07.023 22820262

[34] Müller ML , Peglau L , Moon LDF , et al. Neurotrophin‐3 attenuates human peripheral blood T cell and monocyte activation status and cytokine production post stroke. Exp Neurol. 2022;347:113901. 10.1016/j.expneurol.2021.113901 34688600

[35] Sheldrick A , Camara S , Ilieva M , Riederer P , Michel TM . Brain‐derived neurotrophic factor (BDNF) and neurotrophin 3 (NT3) levels in post‐mortem brain tissue from patients with depression compared to healthy individuals—a proof of concept study. Eur Psychiatry. 2017;46:65‐71. 10.1016/j.eurpsy.2017.06.009 29102768

[36] Hao P , Duan H , Hao F , et al. Neural repair by NT3‐chitosan via enhancement of endogenous neurogenesis after adult focal aspiration brain injury. Biomaterials. 2017;140:88‐102. 10.1016/j.biomaterials.2017.04.014 28641124

[37] Gonzalez S , McHugh TLM , Yang T , et al. Small molecule modulation of TrkB and TrkC neurotrophin receptors prevents cholinergic neuron atrophy in an Alzheimer's disease mouse model at an advanced pathological stage. Neurobiol Dis. 2022;162:105563. 10.1016/j.nbd.2021.105563 34838668

[38] Akyol O , Sherchan P , Yilmaz G , et al. Neurotrophin‐3 provides neuroprotection via TrkC receptor dependent pErk5 activation in a rat surgical brain injury model. Exp Neurol. 2018;307:82‐89. 10.1016/j.expneurol.2018.06.002 29883578 PMC8893599

[39] Farhang S , Barar J , Fakhari A , et al. Asymmetrical expression of BDNF and NTRK3 genes in frontoparietal cortex of stress‐resilient rats in an animal model of depression. Synapse. 2014;68(9):387‐393. 10.1002/syn.21746 24753016

[40] Battaglini D , Robba C , Lopes da Silva A , et al. Brain‐heart interaction after acute ischemic stroke. Crit Care. 2020;24(1):163. 10.1186/s13054-020-02885-8 32317013 PMC7175494

[41] Bi W , Wang J , Jiang Y , et al. Neurotrophin‐3 contributes to benefits of human embryonic stem cell‐derived cardiovascular progenitor cells against reperfused myocardial infarction. Stem Cells Transl Med. 2021;10(5):756‐772. 10.1002/sctm.20-0456 33529481 PMC8046156

[42] Lai BQ , Che MT , Du BL , et al. Transplantation of tissue engineering neural network and formation of neuronal relay into the transected rat spinal cord. Biomaterials. 2016;109:40‐54. 10.1016/j.biomaterials.2016.08.005 27665078

[43] Yang Y , Wei F , Wang J , et al. Manganese modifies Neurotrophin‐3 (NT3) and its tropomyosin receptor kinase C (TrkC) in the cortex: implications for manganese‐induced neurotoxicity. Food Chem Toxicol. 2020;135:110925. 10.1016/j.fct.2019.110925 31676349

[44] Andersen M , Andelius TCK , Pedersen MV , Kyng KJ , Henriksen TB . Severity of hypoxic ischemic encephalopathy and heart rate variability in neonates: a systematic review. BMC Pediatr. 2019;19(1):242. 10.1186/s12887-019-1603-7 31324176 PMC6639904

[45] Cristofaro B , Stone OA , Caporali A , et al. Neurotrophin‐3 is a novel angiogenic factor capable of therapeutic neovascularization in a mouse model of limb ischemia. Arterioscler Thromb Vasc Biol. 2010;30(6):1143‐1150. 10.1161/atvbaha.109.205468 20360537 PMC2881585

[46] Morelli C , Castaldi L , Brown SJ , et al. Identification of a population of peripheral sensory neurons that regulates blood pressure. Cell Rep. 2021;35(9):109191. 10.1016/j.celrep.2021.109191 34077727 PMC8187988

